# Dental Department of Vanderbilt University

**Published:** 1882-05

**Authors:** 


The third annual commencement.

### DENTAL DEPARTMENT OF VANDERBILT UNIVERSITY.

The third annual commencement.

The number of matriculates for the session was seventy-three.

The degree of D.D.S. was conferred on the following persons by Dr. Garland, Chancellor of the University:

| NAME. | RESIDENCE. | NAME. | RESIDENCE. |
|---|---|---|---|
| Thomas M. Allen | Alabama. | Thomas H. Lipscourt | Tennessee. |
| James A. Barr | Kentucky. | Marques J. Lunquest | Alabama. |
| William J. Flanders | Georgia. | John H. Magruder | Louisiana. |
| William A. Flemister | Georgia. | Francis H. McAnnally | Alabama. |
| James W. Hambright | Georgia. | John M. Powell | Texas. |
| Stephen R. Jordan | Georgia. | D. R. Stubblefield, M.D | Tennessee. |
| Wm. W. Kemper, M.D. | Missouri | William J. Tillett | Tennessee. |

